# Methicillin-Resistant Staphylococcus aureus Spinal Epidural Abscess: Local and Systemic Case Management

**DOI:** 10.7759/cureus.22831

**Published:** 2022-03-03

**Authors:** Abdurrahman F Kharbat, Cameron T Cox, Amanda Purcell, Brendan J MacKay

**Affiliations:** 1 Orthopedic Surgery, Texas Tech University Health Sciences Center, Lubbock, USA; 2 Orthopedic Surgery/Hand and Microvascular Surgery, Texas Tech University Health Sciences Center, Lubbock, USA

**Keywords:** differential diagnosis of spinal epidural abscess, treatment of spinal epidural abscess, musculoskeletal infection, decompression of spinal epidural abscess, methicillin-resistant staphylococcus aureus

## Abstract

Spinal epidural abscess (SEA) is a rare condition with complex pathophysiology and highly variable clinical presentation. While it is known to cause focal peripheral nerve symptoms such as muscle weakness, paresthesia, or pain, these are typically accompanied by complaints of back or spine pain and systemic symptoms indicative of infection. In our case, a 53-year-old male initially presented with unilateral pain and swelling in his right hand, with no fever at presentation and no complaints of back pain. Blood culture confirmed methicillin-resistant* Staphylococcus aureus* (MRSA)for which he was given vancomycin*. *The patient later endorsed back pain and diagnostic imaging revealed a spinal epidural abscess spanning the T5-T9 vertebrae. The abscess was drained, and vancomycin was placed in the subfascial and epifascial compartments. The hand was debrided in the same operation and showed no gross purulence. Two days after the procedure, intraoperative cultures remained negative, and the patient was subsequently managed with daptomycin.

## Introduction

In the management of a patient with a spinal epidural abscess (SEA), case presentation must be applied against an understanding of the complexity of disease pathophysiology in order to establish the best steps for patient care. SEA is a historically uncommon and challenging disease with insidious presentation and grave consequences if diagnosis is delayed or missed altogether [1]. It has recently demonstrated a five-fold increase in incidence, highlighting the importance of renewed attention to the variability of SEA presentations [2].

The recent increase in incidence could be attributed to a myriad of factors including increased age, immunosuppressive comorbidities (such as diabetes, end-stage renal disease, and malignancy), intravenous drug use, and spinal procedures [3]. Moreover, a heightened cognizance of SEA on differentials and the use of advanced imaging (MRI and CT) may be contributing to the increasing rates of diagnosis. *Staphylococcus aureus* from skin infections and furuncles is considered to be one of the most common etiologies of SEA, spreading to the spinal epidural space in variable fashion [4,5]. Although previously thought to be a rare etiology [5], *Staphylococcus aureus* from oral flora was demonstrated to be the source of infection in 25% of cases in a modern prospective review, elucidating a transforming understanding of the etiology of SEA [6].

Spinal epidural space is a potential space between the dura mater and vertebral wall that contains fat and a prominent network of blood vessels. Within this space, infection may arise through hematogenous dissemination or contiguous spread from adjacent infections such as osteomyelitis, discitis, or paraspinal abscess [3]. As with the variability in presentation, the pathophysiology of disease is different from one case to the next, with cases producing a myriad of symptoms due to different plausible etiologies [7]. Some of the symptoms of SEA may arise from the expansion of bacterial purulence, which causes compression of nervous structures and results in systemic symptoms and pain, progressing to spinal cord and nerve root dysfunction [3,5]. The efficacy of surgical decompression in treating SEA symptoms supports this mechanism of disease [8]. Postmortem studies have indicated that symptoms may also arise from thrombosis or thrombophlebitis of arteries or veins in the epidural space that produce ischemic infarction of the spinal cord [3,5].

In the care of a patient with SEA, careful attention must be given to all elements of the clinical presentation in order to understand the etiology and mechanism of disease and best strategize the patient’s medical and/or surgical management [9]. Here, we describe a unique case of SEA in which a patient initially presented with pain and swelling in the right hand without a known causative event or chronic condition.

## Case presentation

A 53-year-old male presented to the emergency center with pain and swelling in his right hand for 10 days. He was previously diagnosed at a separate freestanding emergency center with cellulitis and given a prescription for cefdinir antibiotics but chose not to get the prescription filled. The patient denied trauma or injury and stated that he had not experienced any issues with his hand prior to the onset of his current symptoms. The pain in his right hand was exacerbated with movement and alleviated with immobilization. He endorsed a subjective fever, though a temperature of 99.6°F was recorded in the clinic.

**Figure 1:**
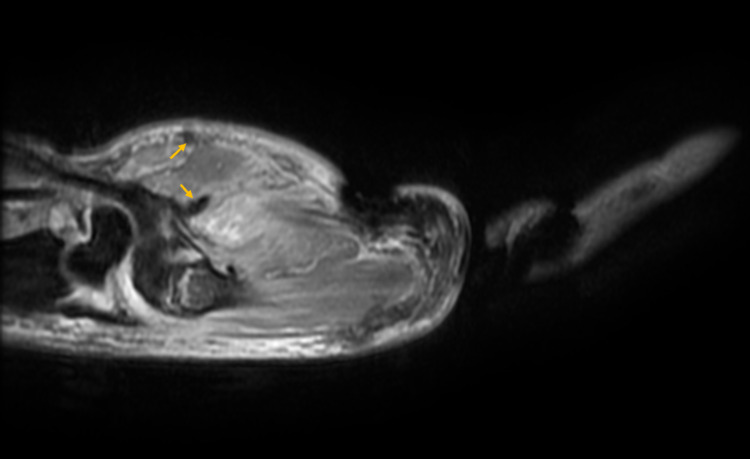
MRI showing small, non-enhancing lesions (arrows) in the thenar musculature and edema in the metacarpals and wrist.

The patient’s right hand demonstrated global swelling and tenderness to palpation, and he was unable to make a composite fist due to swelling and pain. Motor function was otherwise intact to muscles of the hand and forearm. Light touch sensation was intact in all nerve distributions of the right upper extremity. The patient’s significant past medical history included pre-diabetes mellitus, hypertension, and benign prostatic hyperplasia. Magnetic resonance imaging (MRI) was performed for the upper extremity (UE) which showed nonspecific edema in the metacarpals and wrist, with small non-enhancing fluid signals concerning abscesses within the thenar musculature of the right hand (Figure 1).

**Figure 2:**
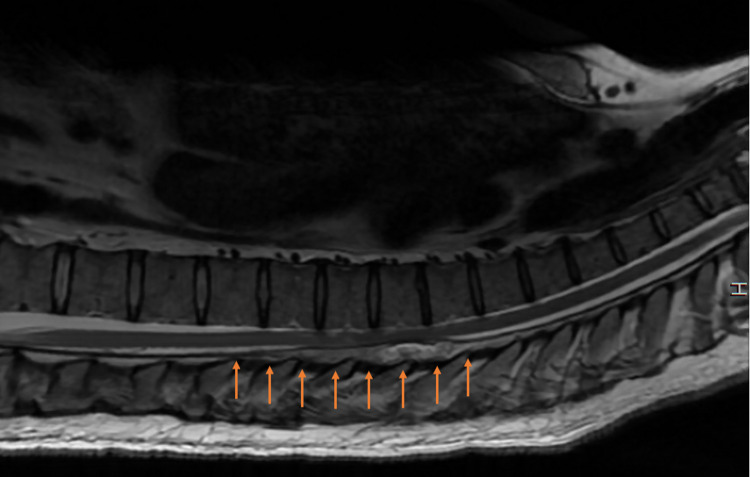
MRI showing abnormal enhancement (arrows) in the dorsal epidural space at T5-T9 levels.

A complete blood count and culture were obtained which revealed elevated white blood cells and platelets as well as elevated granulocyte percentage. He was started on empiric antibiotic management with vancomycin, after which his blood culture grew methicillin-resistant *Staphylococcus aureus* (MRSA). Blood cultures taken two days after initiation of vancomycin were negative. The patient was given one dose of ceftriaxone and subsequently one dose of Zosyn. Upon further questioning, the patient endorsed concomitant back pain and no urine output for four days. Given these findings and the lack of a known causative event for pain in his right hand, MRI imaging was ordered for the lumbar spine which revealed a SEA spanning T5-T9 (Figure 2). 

Four days after presentation to our emergency center (two weeks after symptom onset), the patient was taken to the operating room (OR) for drainage of his spinal abscess. Given that his hand pain and swelling had not improved with antibiotics, irrigation and debridement of the right hand was planned at the same time. The T5-T9 vertebrae were exposed, and the laminae were removed as a single piece. Upon removal, purulent fluid was encountered which was sent for culture. Early phlegmon formation was also present and was dissected away from the underlying dura. The wound was then irrigated, and vancomycin powder was placed in the subfascial compartment. Given that the tissue was hyperemic, a round drain was placed below the fascia and layered closure was performed with additional vancomycin powder placed in the epifascial compartment. After his neurosurgical procedure, the patient was flipped supine and irrigation and debridement of his right hand was performed down to the level of bone. No gross purulence was observed during the procedure.

Transthoracic echocardiogram (TTE) was performed five days after presentation and infective endocarditis was ruled out. Since cultures drawn two days after the presentation (one day after vancomycin treatment) were continuing to return negative, lack of persistent bacteremia was established. The patient was thereafter managed with daptomycin. At nine months postoperatively, the patient was ambulating with a cane and progressing as expected with physical therapy.

## Discussion

As previously indicated, SEA is a rare condition with variable and complex etiology and mechanism of disease [6,10]. If diagnosis is delayed or missed altogether, SEA can progress to severe permanent defects in nervous structures [2,11,12]. Idiopathic back pain has historically been a cause for suspicion of spinal involvement when weakness and/or pain in the extremities present without acute injury or known chronic conditions [3,12].

In our case, the patient did not initially complain of back pain, and the clinical presentation was further complicated by the presence of global swelling of the right hand. While it is uncommon for visible inflammation to develop around a peripheral nerve distribution without structural injury to the peripheral structures, it is plausible that neuropathic pain signals could trigger neurogenic inflammation [13,14]. It is more likely that our patient developed bacteremia secondary to right hand cellulitis which ultimately spread to cause an epidural abscess. 

The discovery of systemic symptoms (obscure back aching and reduced urine output) pushed the diagnosis away from an isolated upper extremity issue, such as radiculopathy, and raised suspicion of SEA. By applying a multi-pronged approach that was able to neutralize the threat of MRSA bacteremia while also ensuring expedited surgical decompression of the SEA and irrigation/debridement of the osteomyelitis infected right hand, our patient was able to recover effectively and with no delay of care.

## Conclusions

The case we present adds to a growing body of literature assessing the clinical presentation, diagnosis, and treatment of SEA. Given the high variability of presentation, unique cases are valuable additions to the diagnostic algorithm in this patient population. The outcome of our case suggests that SEA should be considered as a potential causative agent when there are findings of unilateral peripheral pain and swelling of unknown origin.
